# Boosting the Li-O_2_ pouch cell beyond 860 Wh kg^−1^ with an O_2_-enriched localized high-concentration electrolyte

**DOI:** 10.1093/nsr/nwaf059

**Published:** 2025-02-20

**Authors:** Zhang Wen, Yiwen Liu, Kaiwen Li, Sixie Yang, Haoshen Zhou, Ping He

**Affiliations:** Center of Energy Storage Materials & Technology, College of Engineering and Applied Sciences, Jiangsu Key Laboratory of Artificial Functional Materials, National Laboratory of Solid-State Microstructures and Collaborative Innovation Center of Advanced Microstructures, Nanjing University, Nanjing 210093, China; Center of Energy Storage Materials & Technology, College of Engineering and Applied Sciences, Jiangsu Key Laboratory of Artificial Functional Materials, National Laboratory of Solid-State Microstructures and Collaborative Innovation Center of Advanced Microstructures, Nanjing University, Nanjing 210093, China; Center of Energy Storage Materials & Technology, College of Engineering and Applied Sciences, Jiangsu Key Laboratory of Artificial Functional Materials, National Laboratory of Solid-State Microstructures and Collaborative Innovation Center of Advanced Microstructures, Nanjing University, Nanjing 210093, China; Center of Energy Storage Materials & Technology, College of Engineering and Applied Sciences, Jiangsu Key Laboratory of Artificial Functional Materials, National Laboratory of Solid-State Microstructures and Collaborative Innovation Center of Advanced Microstructures, Nanjing University, Nanjing 210093, China; School of Materials Science and Intelligent Engineering, Nanjing University, Suzhou 315163, China; Center of Energy Storage Materials & Technology, College of Engineering and Applied Sciences, Jiangsu Key Laboratory of Artificial Functional Materials, National Laboratory of Solid-State Microstructures and Collaborative Innovation Center of Advanced Microstructures, Nanjing University, Nanjing 210093, China; Center of Energy Storage Materials & Technology, College of Engineering and Applied Sciences, Jiangsu Key Laboratory of Artificial Functional Materials, National Laboratory of Solid-State Microstructures and Collaborative Innovation Center of Advanced Microstructures, Nanjing University, Nanjing 210093, China

**Keywords:** lithium-oxygen battery, pouch cell, electrolyte, high specific energy, thick electrode

## Abstract

Lithium-oxygen batteries (LOBs) are considered to be the next generation of high-specific-energy storage devices. To improve the practical specific energy, LOBs typically require thick cathode electrodes to achieve higher areal capacity. However, due to the inefficient O_2_ diffusion within the electrolyte-flooded thick cathodes, the practical discharge capacity of LOBs is significantly lower than their ultra-high theoretical value. Herein, we propose a strategy to solve the problem of limited O_2_ diffusion in the thick cathodes of LOBs by applying an O_2_-enriched localized high-concentration electrolyte (LHCE). With a thick cathode (10 mg cm^−2^), LOBs based on this O_2_-enriched LHCE deliver impressive discharge capacities of 50.4, 27.1 and 20.3 mAh cm^−2^ at current densities of 0.1, 0.3 and 0.5 mA cm^−2^, respectively. The discharge product Li_2_O_2_ is homogeneously distributed within the thick cathode due to the enhanced O_2_ diffusion conferred by the O_2_-enriched LHCE, indicating that the product storage space of the thick cathode is more efficiently utilized. Besides, the O_2_-enriched LHCE-based LOBs derive a stable solid electrolyte interphase to protect the Li anode from O_2_ corrosion. Additionally, a pioneering primary Li-O_2_ pouch cell with the O_2_-enriched LHCE achieves an exceptional specific energy of 860.6 Wh kg^−1^ (based on the total pouch cell weight), providing a promising technical pathway for the practical application of LOBs.

## Teaser

This work presents an O_2_-enriched electrolyte that promotes the transport of O_2_ within the cathode to achieve an ultra-high specific energy of the Li-O_2_ pouch cell.

## INTRODUCTION

Lithium-oxygen batteries (LOBs) with an ultra-high theoretical specific energy of 3500 Wh kg^−1^ are generally recognized as the next generation of high-specific-energy batteries. Research on LOBs has brought several breakthroughs in catalysts, electrolytes, electrochemical reaction mechanisms, etc. [1–4]. Nevertheless, most previous work has been based on laboratory-level cells (Swagelok or coin-type cells) with excess electrolyte, surplus lithium metal anode and low cathode loading. This approach ignores many factors that need to be considered to achieve practical high-specific-energy batteries. Therefore, future research should be directed toward the development of Li-O_2_ pouch cells for practical applications. Notably, while many studies have focused on the design of rechargeable LOBs, ultra-high-specific-energy primary LOBs remain indispensable for applications in drones, the military and other extreme environments where recharging is not feasible.

The design of high-specific-energy pouch cells requires a greater focus on areal specific capacity (mAh cm^−2^), rather than merely considering specific capacity based on cathode material weight (mAh g^−1^_cathode_). The hard-to-break areal capacity limit undermines the supposed high-specific-energy advantages of LOBs and hinders their practical application [5,6]. LOBs using thick cathodes with high loading are expected to increase the discharge areal specific capacity. However, addressing the problem of limited O_2_ diffusion in the thick porous electrodes of LOBs seems more challenging, especially since the electrodes are flooded with electrolyte during battery operation. The environment of porous electrodes in LOBs represents the reaction site and storage space for the solid product Li_2_O_2_, and its loading signifies the theoretical upper limit of the capacity. The actual discharge capacity in a thick electrode is mainly limited by the restriction of O_2_ transport. On the one hand, unlike conventional thin electrodes, the O_2_ diffusion process in thick electrodes is determined by several interrelated factors, including thickness, channel size and channel structure. On the other hand, the gradual deposition of solid Li_2_O_2_ during the discharge process leads to clogging of the pores, further reducing the O_2_ diffusion channels. The above issues lead to obvious heterogeneous electrochemical reactions along the depth direction of the thick electrodes, resulting in insufficient utilization of the active area and pore space of the thick electrodes, and an apparent shortfall in the actual specific energy.

Due to its highly reductive nature, the Li metal anode faces many critical issues during the electrochemical reaction of LOBs [7]. The effect of O_2_ and reactive oxygen species crossover in LOBs further complicates the scenario. Researchers have generally ignored these problems by using significant excess Li metal or employing LiFePO_4_ as the anode [8]. However, the construction of high-specific-energy Li-O_2_ pouch cells requires limited usage of Li metal, making the issue of anode parasitic reactions more worthy of attention. Researchers have found that in high-concentration electrolytes (HCEs), the lowest unoccupied molecular orbital (LUMO) energy of the electrolyte shifts from the solvent dominant to the salt dominant, promoting the formation of a salt-derived solid electrolyte interphase (SEI) [9,10]. In fact, the addition of an inert diluent to form localized high-concentration electrolyte (LHCE) reduces the viscosity and cost of HCE while forming a similar SEI to HCE [11,12]. Therefore, the rationally designed LHCE has the potential to suppress the Li metal corrosion by O_2_ and reactive oxygen species.

**Figure 1.**
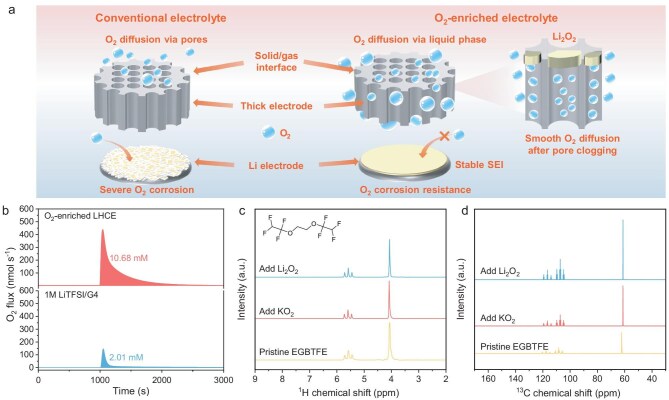
(a) Schematic illustration of the modification for O_2_ diffusion through thick cathodes of LOBs by O_2_-enriched LHCE. (b) Online mass spectrometry tests for the solubility of O_2_ in various electrolytes. (c) ^1^H-nuclear magnetic resonance (NMR) and (d) ^13^C-NMR spectra of various electrolytes before and after adding KO_2_ and Li_2_O_2_.

In this work, we propose a strategy to boost the specific energy of LOBs by applying a LHCE with high O_2_ solubility (Fig. 1a). Inspired by perfluorocarbon (PFC) artificial blood [13,14], a fluorinated ether of ethylene glycol bis(1,1,2,2-tetrafluoroethyl) ether (EGBTFE) is used as a multifunctional diluent of LHCE. Benefiting from the abundant C–F bonds of EGBTFE, this LHCE has the ability to dissolve sufficient O_2_ to overcome the challenge of O_2_ diffusion limitations within the thick cathodes of LOBs. In addition, the inorganic-rich SEI formed in LHCE protects the Li metal anode from corrosion by O_2_. Besides, EGBTFE as a diluent reduces the electrolyte viscosity, improves the solid/liquid interface wettability, and saves the usage of electrolyte in LOBs. As a result, O_2_-enriched LHCE-based LOBs have exhibited a discharge capacity of 50.4 mAh cm^−2^ at a current density of 0.1 mA cm^−2^, approximately four times higher than that of conventional 1 M LiTFSI/G4-based LOBs (13.4 mAh cm^−2^). The electrode sectioning technique was performed using a cryostat microtome to determine the distribution of Li_2_O_2_ along the perpendicular direction of the discharged cathode. The homogeneous Li_2_O_2_ distribution and the efficient utilization of the cathode pore space further verified that the excellent solubility capability of O_2_ in LHCE contributes to the enhancement of the discharge capacity of the LOBs. Significantly, by designing a double-cathode stacked layer structure, a 3.52-Ah-scale primary Li-O_2_ pouch cell with the O_2_-enriched LHCE achieves a specific energy of up to 860.6 Wh kg^−1^ (based on the total weight of the pouch cell). The successful manufacture of ultra-high-specific-energy primary Li-O_2_ pouch cells shows great potential in drones, the military, aerospace and other extreme environments.

## RESULTS AND DISCUSSION

### The design strategy of O_2_-enriched LHCE

Due to their extremely high O_2_ solubility conferred by the abundant strongly polar C–F bonds, PFCs have been successfully applied in artificial blood [13,14]. Inspired by this, we attempt to introduce fluorinated organic solvents into the LOB electrolyte to boost the O_2_ solubility, thereby increasing the areal discharge capacity and specific energy. However, PFCs hardly dissolve lithium salts and tend to be immiscible with common LOB electrolytes [15]. Fluorinated ethers attract our interest since they also have many C–F bonds and they are widely used as diluents for LHCE in lithium-ion batteries, demonstrating their miscibility with common ethers. As shown in Table S1, considering volatility, density, flammability and cost factors, EGBTFE was selected as the multifunctional diluent for LHCE. Subsequently, the design of salt-solvent clusters in LHCE was based on lithium bis(trifluoromethanesulfonyl)imide (LiTFSI) as the lithium salt and tetraethylene glycol dimethyl ether (G4) as the solvent, which has shown relatively high stability and reliability for LOBs in previous studies [16,17].

### Physicochemical properties of O_2_-enriched LHCE

The solubility of O_2_ in different electrolytes was measured using an online mass spectrometer [18]. The electrolyte was saturated by O_2_ and then flushed with an Ar flow to expel all O_2_ dissolved in the electrolyte. Subsequently, the released O_2_ was quantified by integrating the mass spectrometry curve. As shown in Fig. 1b, the solubility of O_2_ in 1 M LiTFSI/G4 and EGBTFE-LHCE (with a volume ratio of 3.4 M LiTFSI/G4 to EGBTFE of 4 : 6) is 2.01 mM and 10.68 mM, respectively. The O_2_ solubility in this LHCE exhibits strong competitiveness compared to other LOB electrolytes reported in the published literature (Table S2). Therefore, the EGBTFE diluent-based LHCE is defined as the O_2_-enriched LHCE. In addition, digital photographs showed that the LiTFSI was not soluble in EGBTFE (Fig. S1), meeting the basic criteria for serving as a diluent. To confirm this, Raman spectra were further conducted to investigate the solvation structure of different electrolytes (Fig. S2). The O_2_-enriched LHCE exhibited similar Raman shift peaks to those of 3.4 M HCE, indicating that the EGBTFE diluent retains the HCE solvation structure. The ionic conductivity at room temperature of the EGBTFE diluent-based LHCE with different compositions is shown in Fig. S3. The addition of EGBTFE diluent reduces the electrolyte viscosity and thus increases the ionic conductivity of the HCE to 0.56 mS cm^−1^, which helps to accelerate the electrochemical reaction kinetics.

During discharge/charge cycling, LOBs produce complex reactive oxygen intermediates such as singlet oxygen (^1^O_2_), superoxo (O_2_^−^) and peroxo (O_2_^2−^) [19–21]. Therefore, the chemical stability of the electrolyte against these reactive oxygen intermediates is particularly critical. As shown in Fig. 1c and d, after soaking with KO_2_ and Li_2_O_2_ for several days, both the ^1^H and ^13^C-nuclear magnetic resonance (NMR) spectra of EGBTFE showed no peaks associated with side reaction products. Besides, the ultraviolet-visible (UV-vis) peak intensities of KO_2_ and Li_2_O_2_ were almost identical before and after addition, indicating that O_2_^−^ and O_2_^2−^ have a negligible effect on the EGBTFE (Fig. S4). As depicted in Fig. S5, the contact angle of the O_2_-enriched LHCE on Li metal, polyethylene (PE) separator, and cathode is 20.8°, 24.2° and 27.9° respectively. The relatively low contact angle indicates that the EGBTFE diluent improves the wettability of highly concentrated electrolytes, potentially reducing the amount of electrolyte required for battery operation and increasing the specific energy [22,23]. In addition, the O_2_-enriched LHCE is non-flammable due to the flame-retardant capability of the EGBTFE diluent, which has great significance for improving the safety of Li-O_2_ batteries (Fig. S6).

### Electrochemical properties of LOBs

**Figure 2.**
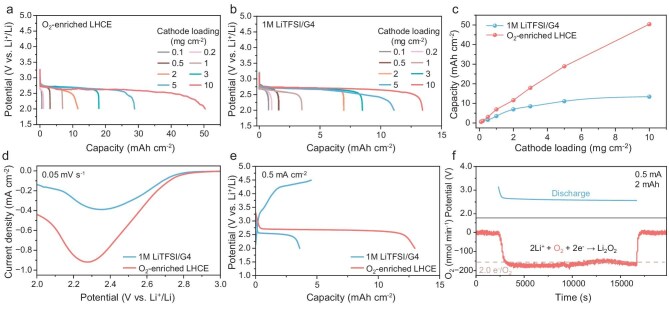
(a) Full discharge curves of the LOBs with different cathode loading based on O_2_-enriched LHCE. (b) Full discharge curves of the LOBs with different cathode loading based on 1 M LiTFSI/G4. (c) Relationship between discharge areal specific capacity and cathode loading of LOBs. (d) Linear sweep voltammetry (LSV) curves of LOBs with different electrolytes within O_2_ atmosphere. (e) Full discharge-charge curves of LOBs with a 5 mg cm^−2^ loaded cathode. The first cycle is with the 1 M LiTFSI/G4 and the second cycle is with the O_2_-enriched LHCE. (f) DEMS analysis of the O_2_-enriched LHCE-based LOB during discharge operation at a current of 0.5 mA.

Figure 2a and b show the full discharge profiles of LOBs with O_2_-enriched LHCE and 1 M LiTFSI/G4 at a current density of 0.1 mA cm^−2^ and a cutoff voltage of 2 V, respectively. The relationship between the discharge areal capacity and the cathode loading is summarized in Fig. 2c. When the cathode loading is less than 0.5 mg cm^−2^, LOBs with two of the above electrolytes exhibit a similar discharge areal capacity. This indicates that O_2_ diffusion is not the determining factor in the discharge capacity of LOBs when using thin electrodes. Interestingly, the discharge areal capacity of LOBs with O_2_-enriched LHCE increases approximately in proportion to the rise of the cathode loading. With a cathode loading of 10 mg cm^−2^, the areal capacity reaches 50.4 mAh cm^−2^. As for LOBs with 1 M LiTFSI/G4, the areal capacity hardly exceeds 13.4 mAh cm^−2^, even if the loading is elevated to 10 mg cm^−2^. The large difference in discharge capacity of LOBs with thick electrodes indicates that the efficient diffusion of O_2_ through the liquid phase allows for a sufficient supply of O_2_ to the pores deep within the electrodes. In addition, the discharge areal capacity of the LOBs can also reach 27.1 mAh cm^−2^ and 20.3 mAh cm^−2^ at a higher current density of 0.3 mA cm^−2^ and 0.5 mA cm^−2^ (Figs S7 and S8). As shown in Fig. 2d, both 1 M LiTFSI/G4 and O_2_-enriched LHCE-based LOBs exhibit a cathodic peak related to the formation of Li_2_O_2_ during the O_2_ reduction reaction (ORR). Nevertheless, the ORR peak current of the O_2_-enriched LHCE-based LOB is up to 0.92 mA cm^−2^, much higher than that of the 1 M LiTFSI/G4-based one (0.39 mA cm^−2^). The large ORR peak current indicates that LOBs with O_2_-enriched LHCE have sufficient O_2_ supply to accelerate the reaction kinetics. To further demonstrate the benefits of the liquid phase O_2_ transport for thick electrodes, a 10 mg cm^−2^ loaded cathode was first discharge-charged for one cycle at 0.5 mA cm^−2^ with the 1 M LiTFSI/G4. A discharge capacity of only 3.6 mAh cm^−2^ was obtained (Fig. 2e). Afterwards, the cathode was carefully washed with dimethoxyethane (DME), dried and then reassembled with the O_2_-enriched LHCE. Surprisingly, the discharge capacity of the LOB reassembled with the O_2_-enriched LHCE increased to 12.9 mAh cm^−2^ and the discharge voltage plateau rose to ∼2.67 V. It is reasonable to infer that an adequate O_2_ supply by O_2_-enriched LHCE allows the thick electrodes to carry out sufficient electrochemical reactions at even greater depths away from the gas/solid interface, resulting in a significant reduction of the local current density and hence decreasing the polarization.

### Electrochemical reaction mechanism and product analysis

The O_2_ consumption and evolution during the discharge and charge process of the O_2_-enriched LHCE-based LOBs were quantitatively analyzed by differential electrochemical mass spectrometry (DEMS). During discharge, the O_2_ consumption gradually increased and stabilized at a charge-to-mass ratio close to 2.0 e^−^/O_2_, corresponding to the conventional two-electron reaction based on 2 Li + O_2_ → Li_2_O_2_ (Fig. 2f). The overall charge-to-mass ratio for the discharge process was calculated as 1.92. The O_2_ evolution during the charge process was lower than the expected 2.0 e^−^/O_2_, possibly due to the complex parasitic reactions during the charging process (Fig. S9). In addition, UV-vis/TiOSO_4_ spectroscopic titration was employed to quantify the Li_2_O_2_ yield (Fig. S10). As shown in Fig. S11, when LOBs were discharged to 1mAh cm^−2^, a Li_2_O_2_ yield of 75.9% was obtained with the O_2_-enriched LHCE, slightly lower than that with 1 M LiTFSI/G4 (82.3%, in agreement with previous research) [24,25]. When LOBs with the O_2_-enriched LHCE were discharged to 20 mAh cm^−2^, the Li_2_O_2_ yield decreased to 42.4%. X-ray photoelectron spectroscopy (XPS) of the discharged cathode reveals the presence of traces of Li_2_CO_3_ parasitic products (Fig. S12). The corrosion of Li_2_O_2_ due to prolonged exposure to electrolyte and carbon electrodes may account for the decrease in yield. The declining Li_2_O_2_ yield after high-capacity discharge also contributed to the inadequate O_2_ evolution during charging monitored by the DEMS.

### Distribution of discharge products within the cathodes

**Figure 3.**
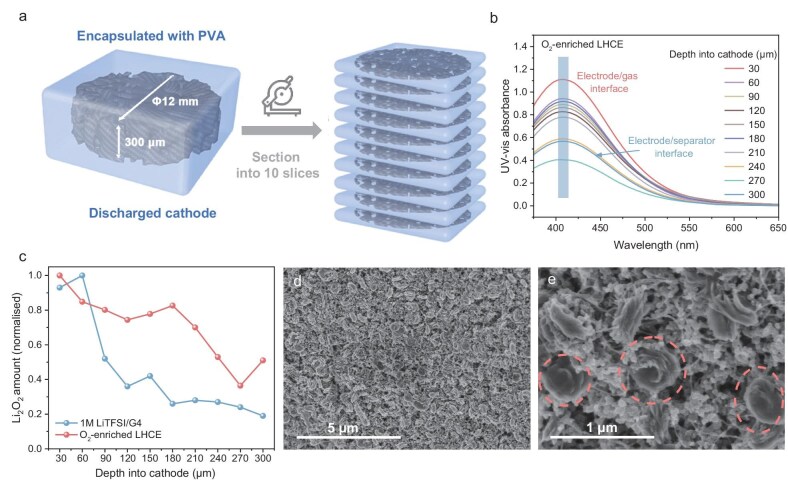
(a) Schematic illustration of the discharged electrode sectioning process. (b) The UV-vis spectra of the TiOSO_4_ solutions with the discharged cathode microtomed to 30 μm slices. (c) Distribution of Li_2_O_2_ across the thick electrodes discharged in 1 M LiTFSI/G4 and O_2_-enriched LHCE. (d and e) SEM images of the discharged cathode obtained from LOB with O_2_-enriched LHCE.

Quantitative analysis of the distribution of the discharge product Li_2_O_2_ within the thick cathodes is critical to understanding the mechanism by which O_2_-enriched LHCE contributes to the increase in discharge capacity. Inspired by the biological-tissue-sectioning-technique, we sectioned the discharged cathodes using a cryostat microtome to determine the distribution of Li_2_O_2_ along the perpendicular direction of the cathode (Fig. 3a). Subsequently, each slice of cathode was titrated by UV-vis/TiOSO_4_ (Fig. 3b and Fig. S13). As shown in Fig. 3c, after discharging with conventional 1 M LiTFSI/G4 electrolyte, Li_2_O_2_ was mainly distributed within 90 µm near the cathode/gas interface. The amount of Li_2_O_2_ inside the electrode was significantly lower than near the cathode/gas interface. This indicates that when thick porous electrodes are used as LOB cathodes, the discharged products grow preferentially at the electrode/gas interface where O_2_ is most enriched [26–28]. As the discharge products accumulate, the electrode pores near the cathode/gas interface are further clogged and O_2_ diffusion is impeded, resulting in the pores in the depths not being fully utilized [26,29]. However, the distribution of Li_2_O_2_ is relatively homogeneous within the thick electrode discharged in O_2_-enriched LHCE. Even near the deepest cathode/separator interface, a notable amount of Li_2_O_2_ is observed (Fig. 3c). The difference in Li_2_O_2_ distribution indicates that O_2_ fills the thick electrode pores and can freely diffuse through the O_2_-enriched LHCE even after the surface pores are clogged. The morphology of the discharge products was further characterized using scanning electron microscopy (SEM). The pristine cathode showed a porous and fluffy state (Fig. S14a). In contrast to the membranous discharge products of 1 M LiTFSI/G4-based LOBs [30], the discharge products of O_2_-enriched LHCE-based LOBs exhibited large circular particles (Fig. 3d and e). The circular shape of Li_2_O_2_ facilitates more efficient utilization of the electrode pore space, as opposed to adhering to the electrode surface, which passivates the electrode and impedes further discharge reaction. For the cathode/separator interface, LOBs discharged with 1 M LiTFSI/G4 show trace amounts of membranous discharge products (Fig. S14b). Whereas, the cathode/separator interface of the O_2_-enriched LHCE-based LOBs is distributed with abundant circular particle products, further demonstrating that increasing the O_2_ solubility of the electrolyte facilitates the efficient utilization of the thick cathode (Fig. S14c and d).

### Influence of O_2_-enriched LHCE on Li metal anodes

**Figure 4.**
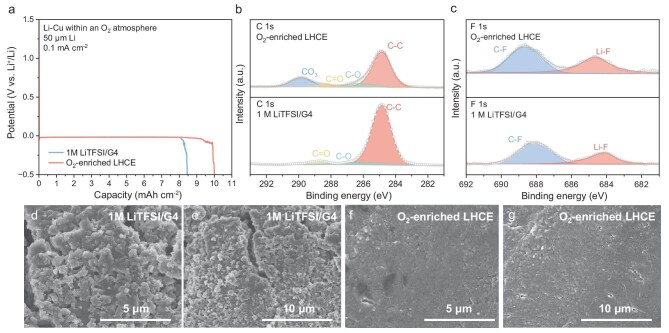
(a) Electrochemical performance of Li-Cu batteries within an O_2_ atmosphere based on different electrolytes with a 50 μm Li anode. (b) C 1s and (c) F 1s XPS spectra of the Li anodes disassembled from the LOBs discharged with different electrolytes. SEM images of Li anodes disassembled from the LOBs discharged with (d and e) 1 M LiTFSI/G4 and (f and g) O_2_-enriched LHCE.

Li metal is the only active material in LOBs that participates in the electrochemical reaction (O_2_ is supplied from outside the battery). However, O_2_ and reactive oxygen species can severely corrode thin Li metals, resulting in the actual capacity being lower than the theoretical capacity. In order to increase the specific energy and reduce the cost of Li-O_2_ pouch cells, it is particularly critical to limit the amount of Li metal anodes. Li-Cu batteries were used to verify the practical discharge capacity of Li metal within an O_2_ atmosphere. As shown in Fig. 4a, the Li-Cu battery with O_2_-enriched LHCE delivers a higher discharge capacity than the battery with 1 M LiTFSI/G4 (the theoretical capacity of a 50 μm thick Li metal is 10.31 mAh cm^−2^). The difference in the practical discharge capacity of Li metal shows that Li metal anodes have different degrees of side reaction in different electrolytes. Therefore, XPS analysis was conducted to investigate the surface chemical state of the Li metal anodes after discharge. The relative proportion of CO_3_ and Li-F groups in the SEI layer discharged with O_2_-enriched LHCE is higher than that of 1 M LiTFSI/G4 (Fig. 4b and c, and Fig. S15). This indicates that the SEI layer with a higher content of Li_2_CO_3_ and LiF effectively stabilizes the Li metal surface and inhibits further corrosion by O_2_ [31,32]. SEM images also showed that the corrosion of Li metal anodes discharged with O_2_-enriched LHCE was obviously lower than that of 1 M LiTFSI/G4 (Fig. 4d–g and Fig. S16). The above results demonstrate that the salt-derived SEI can effectively protect the Li metal anode even though the easier O_2_ shuttling to the Li metal surface caused by the LHCE contains large amounts of O_2_. In the Li-O_2_ pouch cell design, the incorporation of O_2_-enriched LHCE can reduce the amount of Li metal, thereby enhancing specific energy and maintaining cost efficiency.

### Technological exploration of practical Li-O_2_ pouch cells

**Figure 5.**
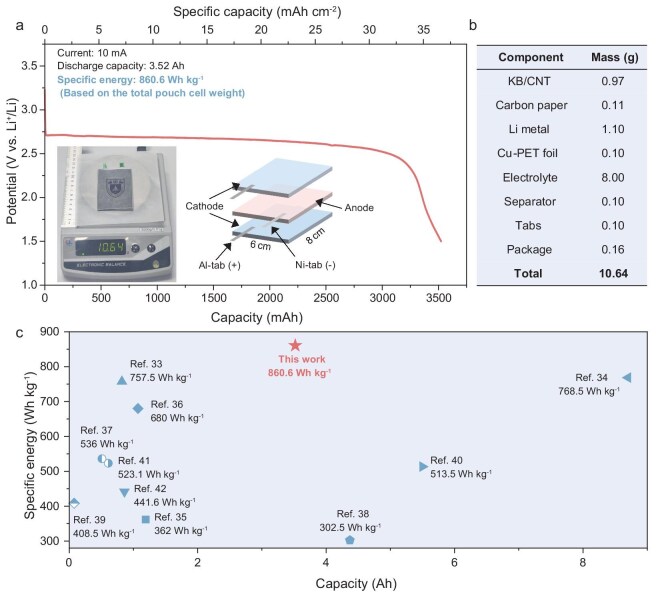
(a) The discharge performance of the Li-O_2_ pouch cell with O_2_-enriched LHCE (inset: digital photograph of the Li-O_2_ pouch cell weighing 10.64 g and schematic illustration of the electrode stacking method). (b) Cell parameters of the Li-O_2_ pouch cell. (c) Comparison of the electrochemical performance of published Li-O_2_ pouch cells [33–42].

Based on the above experiments and discussions, we design a Li-O_2_ pouch cell based on O_2_-enriched LHCE to verify practical applicability. The detailed assembly and test methods for the Li-O_2_ pouch cell can be found in the Methods section of the supplementary data. It can be seen from Fig. 5a that the O_2_-enriched LHCE-based Li-O_2_ pouch cell exhibits a discharge capacity of 3.52 Ah and an areal capacity of 36.7 mAh cm^−2^, indicating that the high discharge capacity of the O_2_-enriched LHCE-based Swagelok-type batteries can be well inherited by the large-sized pouch cells. Based on the total weight of the pouch cell, the specific energy is up to 860.6 Wh kg^−1^. When taking only the weight of the pouch cell core (cathode, anode and electrolyte) into account, the specific energy is even higher at 909.3 Wh kg^−1^. Detailed cell parameters of the Li-O_2_ pouch cell are listed in Fig. 5b and Fig. S17. The discharge product, Li_2_O_2_, is uniformly distributed in different regions of the pouch cell cathode (Fig. S18). It is worth noting that the specific energy of the O_2_-enriched LHCE-based pouch cell exceeds most of the recently reported Li-O_2_ pouch cells, which is calculated based on the total cell weight (Fig. 5c and Table S3) [33–43]. The O_2_-enriched LHCE-based Li-O_2_ pouch cell also demonstrates strong competitiveness compared to other advanced battery systems (Fig. S19 and Table S4) [44–49]. Subsequently, the pressure applied to the Li-O_2_ pouch cell is ∼25.61 kPa, which is measured by a homemade pressure-sensing device with a membrane force-sensitive resistor (Fig. S20) [50]. In addition, the O_2_-enriched LHCE-based Li-O_2_ pouch cell successfully triggered the charging process of a smartphone and illuminated a red LED light board with an ‘NJU’ pattern, as shown in Fig. S21. The successful fabrication of pouch cells not only demonstrates the practical viability of O_2_-enriched LHCE-based LOBs, but also provides valuable insight into the engineering of high-specific-energy devices.

## CONCLUSION

In conclusion, an O_2_-enriched LHCE has been elaborately designed to boost the specific energy of LOBs. Benefiting from the abundant C–F bonds of EGBTFE, this LHCE has the ability to dissolve sufficient O_2_ to overcome the challenge of O_2_ diffusion limitations within the thick cathodes of LOBs. As a result, O_2_-enriched LHCE-based LOBs have exhibited a discharge capacity of 50.4 mAh cm^−2^ at a current density of 0.1 mA cm^−2^, which is much higher than the 13.4 mAh cm^−2^ of the 1 M LiTFSI/G4-based LOBs. In addition, O_2_-enriched LHCE-based LOBs achieve discharge capacities of 27.1 mAh cm^−2^ and 20.3 mAh cm^−2^ at higher rates of 0.3 mA cm^−2^ and 0.5 mA cm^−2^, respectively. A charge-to-mass ratio close to 2.0 e^−^/O_2_ during the discharge process of O_2_-enriched LHCE-based LOBs is obtained by DEMS, and a 75.9% yield of discharge product Li_2_O_2_ is characterized by UV-vis/TiOSO_4_ titration. Combining the electrode sectioning technique and UV-vis/TiOSO_4_ titration, we reveal that the discharge products of O_2_-enriched LHCE-based LOBs are homogeneously distributed in the thick cathodes, and the pore space of cathodes is efficiently utilized. However, the discharge products of conventional 1 M LiTFSI/G4-based LOBs are almost distributed near the cathode/gas interface. O_2_-enriched LHCE-based LOBs derive a Li_2_CO_3_/LiF-rich SEI to protect the Li anode from O_2_ corrosion. Impressively, a 3.52-Ah-scale Li-O_2_ pouch cell with the O_2_-enriched LHCE achieves a specific energy of up to 860.6 Wh kg^−1^(based on the total pouch cell weight) by a double-cathode stacked layer structure. The successful fabrication of ultra-high-specific-energy Li-O_2_ pouch cells promotes primary LOBs as an attractive energy-storage device for drones, the military, aerospace and other extreme environments.

## Supplementary Material

nwaf059_Supplemental_File
