# Supplementary material for: Boosting the Li-O2 pouch cell beyond 860 Wh kg−1 with an O2-enriched localized high-concentration electrolyte
Source: Natl Sci Rev. 2025 Feb 20;12(7):nwaf059. doi: 10.1093/nsr/nwaf059 (PMC12153714; doi:10.1093/nsr/nwaf059)
Supplement: nwaf059_Supplemental_File [file nwaf059_supplemental_file.pdf]

# Supporting Information

## Boosting the Li-$O_2$ pouch cell beyond 860 Wh $kg^{-1}$ by an $O_2$-enriched localized high-concentration electrolyte

Zhang Wen[1], Yiwen Liu[1], Kaiwen Li[1], Sixie Yang[1,2], Haoshen Zhou[1], and Ping He[1,*]

[1]Center of Energy Storage Materials & Technology, College of Engineering and Applied Sciences, Jiangsu Key Laboratory of Artificial Functional Materials, National Laboratory of Solid-State Microstructures and Collaborative Innovation Center of Advanced Microstructures, Nanjing University, Nanjing 210093, China.

[2]School of Materials Science and Intelligent Engineering, Nanjing University, Suzhou 315163, China.

*__Corresponding authors.__ E-mail: pinghe@nju.edu.cn

# MATERIALS AND METHODS

## Materials

Tetraethylene glycol dimethyl ether (G4), lithium bis(trifluoromethanesulphonyl)imide (LiTFSI), lithium hydroxide monohydrate ($LiOH·H_2O$), lithium nitrate ($LiNO_3$), dimethoxyethane (DME), methanol, isopropyl alcohol, and $TiOSO_4$ were purchased from Sigma-Aldrich. Ethylene glycol bis(1,1,2,2-tetrafluoroethyl) ether (EGBTFE) were purchased from Apollo. Ketjen Black (KB), carbon nanotubes (CNT) and polytetrafluoroethylene (PTFE) were achieved from Canrd Technology Co., Ltd. Lithium foils with different thicknesses were obtained from China Energy Lithium Co., Ltd. Carbon paper (CP) were customized by Guangdong Hydrogen Engine New Material Co., Ltd. 4A molecular sieves were soaked in all solvents for 7 days to remove traces of water prior to electrolyte preparation.

## Electrodes preparation, batteries assembly and electrochemical performance tests

Cathodes for Swagelok batteries and pouch cells were prepared by an ultrasonic spraying instrument (XF-10, Suzhou Xifeng Automation Co., Ltd). The cathode precursor ink consisted of KB, CNT, ultrapure water, and isopropyl alcohol. After ultrasonic dispersion, the ink was ultrasonically sprayed on the carbon paper surface at a flow rate of 0.5 mL $min^{-1}$. The loading mass was controlled by the number of passes across a given runway, which was preset in the program. Cathode for slice analysis were prepared by the dry electrode process. The self-supporting electrodes for electrode sectioning were composed of 75 wt.% KB and 25 wt.% PTFE. Their thickness was controlled to 300 μm by a roller press. Anodes for pouch cells were fabricated by rolling Li foils of 200 μm thickness on both sides of Cu-PET composite foils. After rolling, the thickness of the Li foil on a single side was approximately 198 μm.

Swagelok batteries were assembled in an Ar-filled glove box. Pouch cells were assembled in a dry room with dew points below -40℃. To ensure complete electrolyte wetting, the pouch cells were vacuum encapsulated in an aluminum-plastic film for 3 days prior to electrochemical performance tests. After sufficient electrolyte infiltration, the Li-$O_2$ pouch cells were removed from the aluminum-plastic film vacuum bag and then transferred to a test device with adequate gas space for the electrochemical performance testing (Figure S22). Electrolyte-wetted $O_2$ was injected into the glass container at a flow rate of 0.05 L $min^{-1}$ during the test. A gas-permeable

pressurizer was used to apply pressure to the pouch cell during battery testing and the pressure applied to the pouch cells was measured by a homemade pressure sensing device with a membrane force sensitive resistor (MFSR).

Linear sweep voltammetry (LSV) measurements were performed on an electrochemical workstation (CS2350m, Wuhan CorrTest Instruments Corp., Ltd.). Galvanostatic tests were performed on the LAND instrument (Wuhan LAND electronics Co., Ltd). EIS tests were conducted on Solartron1287/1260 with a frequency range from 1 MHz to 0.1 Hz and an amplitude of 5 mV.

**Method of sectioning the cathode**

The discharged electrodes were encapsulated with a commercial polyvinyl alcohol (PVA) liquid adhesive and placed at -30 ℃ to solidify. Then, electrodes were sliced into discs with same thickness using a cryostat microtome (MEV, SLEE medical GmbH). The discs were then added to the appropriate amount of $TiOSO_4$ solution to perform the UV-vis titration test.

**Characterization**

Electrochemical mass spectrometry (DEMS) measurements were performed using a quadrupole mass spectrometer (PrismaPro QMG 250 M2) with a gas flow meter system (ACU10FA, Accu-Flo Meter Service Ltd). Scanning electron microscopy (SEM) was taken on a Hitachi SU8010 to observe morphology and microstructure. Ultraviolet–visible absorption spectra (UV-vis) were recorded using a spectrophotometer (UV-3600 Plus, Shimadzu). Nuclear magnetic resonance (NMR) spectroscopy was performed by Avance III 400MHz (Bruker) to analyze the molecular structures and chemical stability of the electrolyte. The wettability of the electrolyte was tested using a contact angle meter (Dataphysics OCA 20). Raman spectra were measured by a confocal microscope spectrometer (Renishaw inVia).

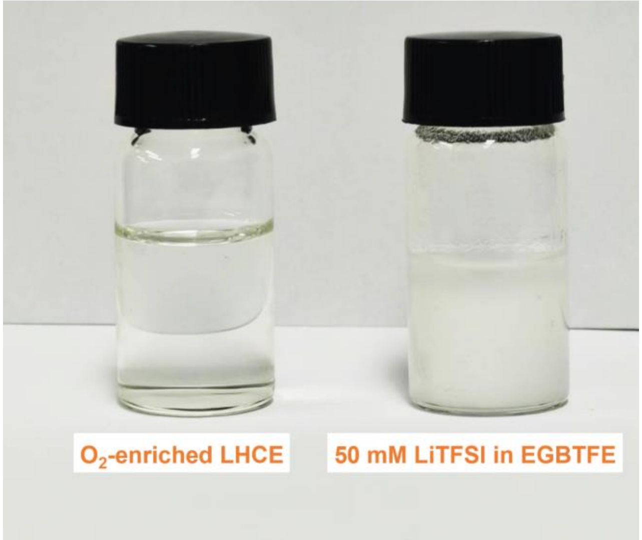


Figure S1. Digital images of the (a) $O_2$-enriched LHCE and (b) 50 mM LiTFSI in EGBTFE. The LiTFSI is not soluble in the EGBTFE.

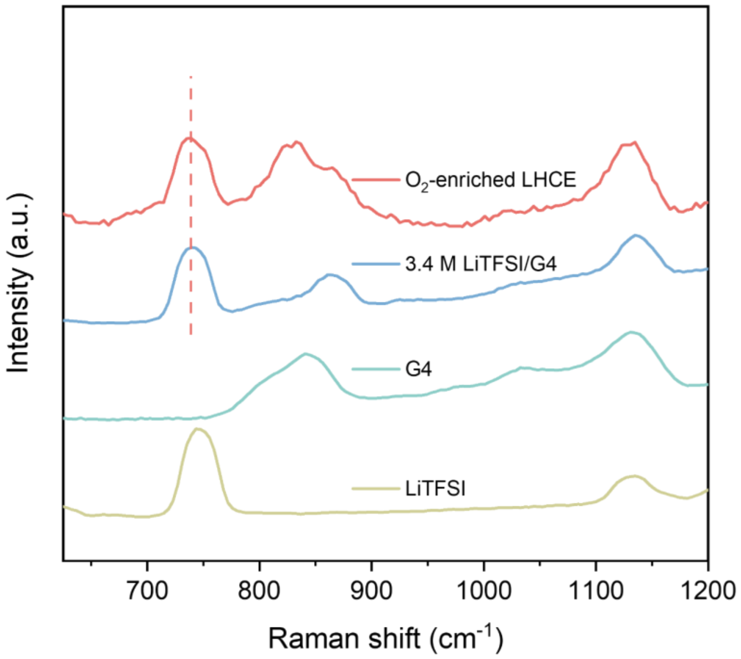


Figure S2. Raman spectra of different electrolytes.

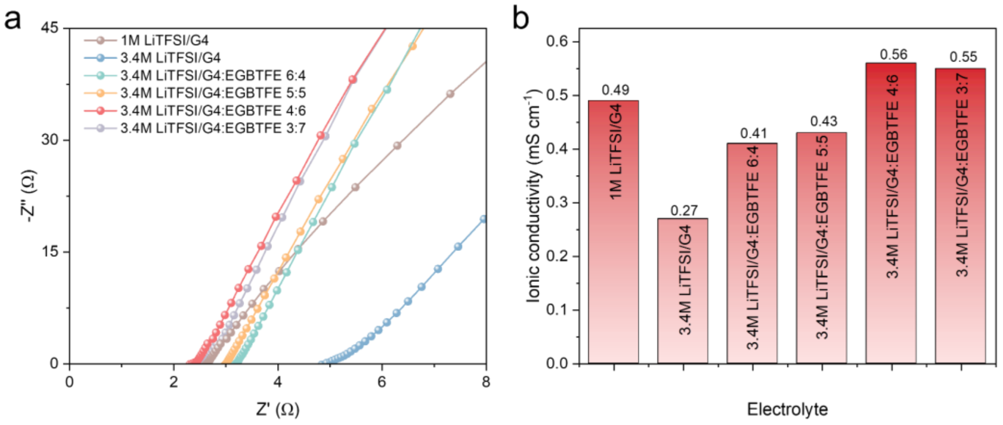


Figure S3. (a) The Nyquist plots of various electrolytes. (b) The ionic conductivity of various electrolytes.

The $Li^+$ conductivity ($\sigma_{Li}$) was calculated by the following equation:

$$\sigma_{Li} = L/(R \times S)$$

Where L is the thickness of the separator, R is the bulk resistance, S is the area of inert electrode (stainless steel). In this work, the thickness of separator is 0.025 mm and area of stainless steel is $1.91 cm^2$ (Φ15.6 mm).

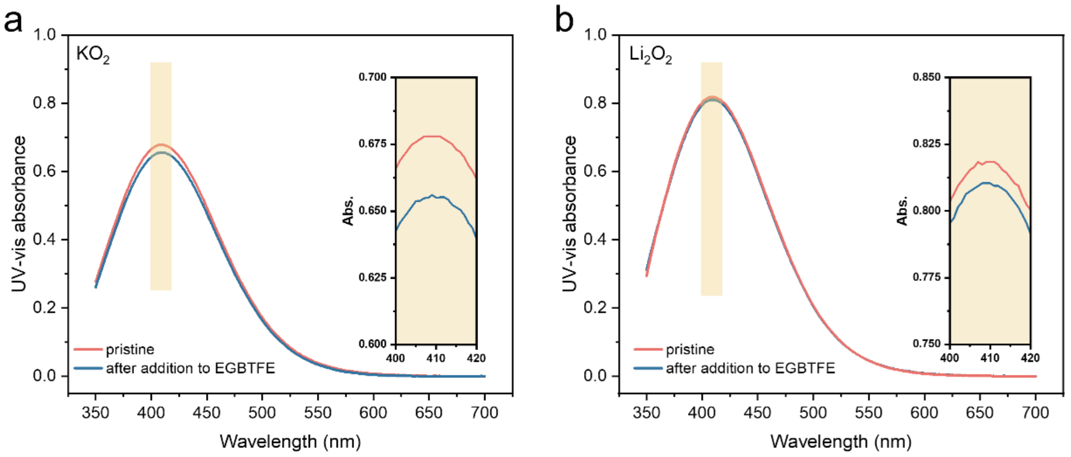


Figure S4. UV-vis spectra of $TiOSO_4$ solution with certain amounts of $KO_2$ (a) and $Li_2O_2$ (b) before and after adding the EGBTFE.

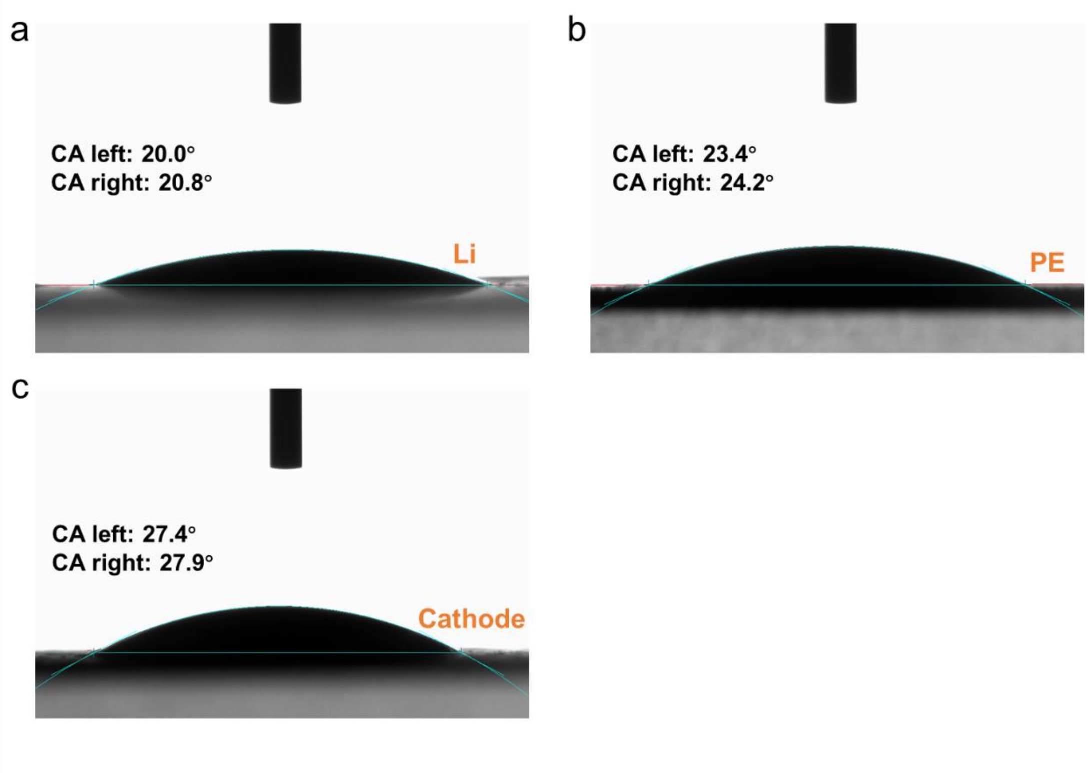


Figure S5. Contact angles of $O_2$-enriched LHCE with Li metal anode (a), PE separator (b), and cathode (c).

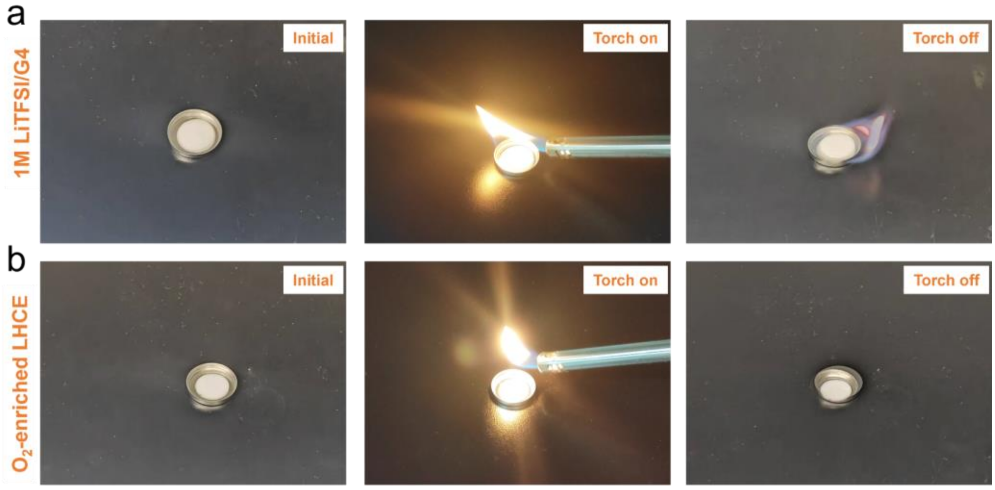


Figure S6. Flammability tests for the (a) 1M LiTFSI/G4 and (b) $O_2$-enriched LHCE.

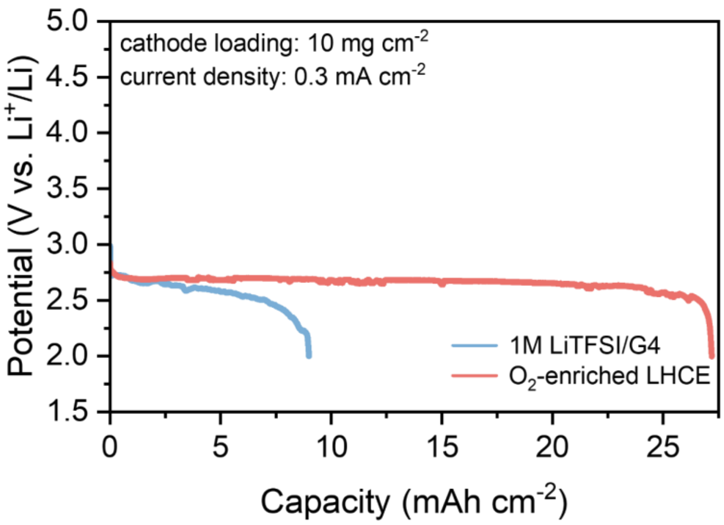


Figure S7. Full discharge curves of the LOBs based on $O_2$-enriched LHCE and 1M LiTFSI/G4 at 0.3 mA $cm^{-2}$.

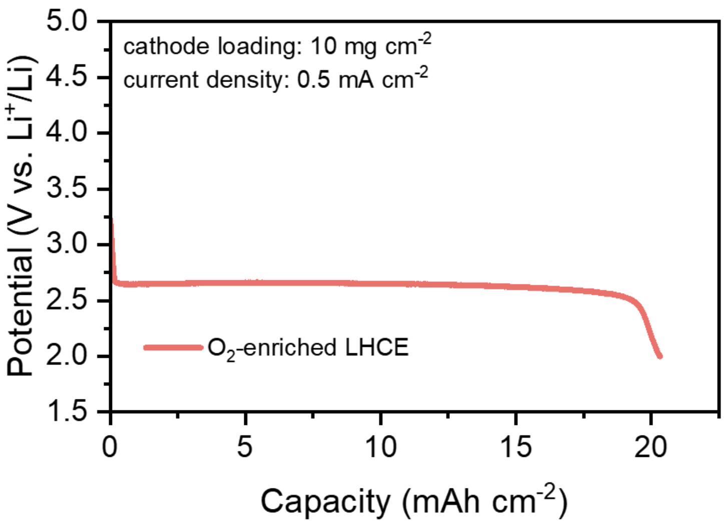


Figure S8. Full discharge curves of the LOBs based on $O_2$-enriched LHCE at 0.5 mA $cm^{-2}$.

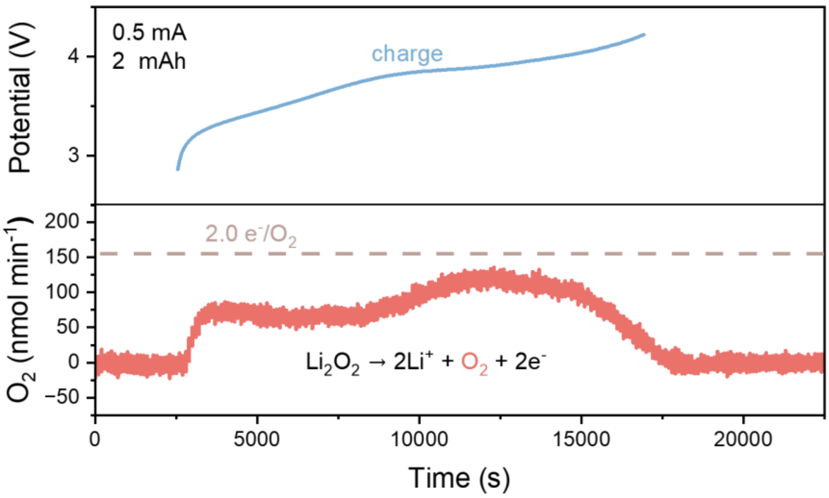


Figure S9. DEMS analysis of the $O_2$-enriched LHCE-based LOB during charge operation at a current of 0.5 mA.

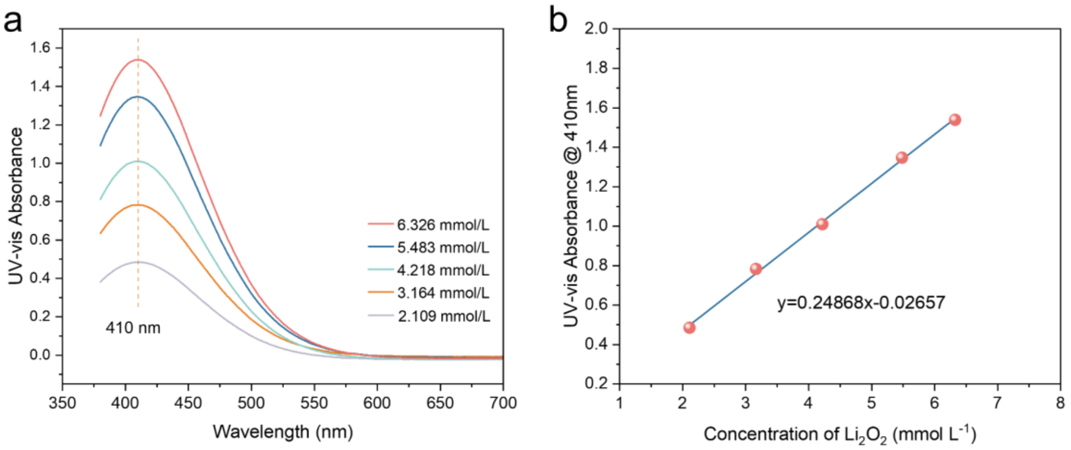


Figure S10. (a) UV-vis spectrum of different $Li_2O_2$ concentrations in $H_2O$. (b) Calibration curve for UV-vis absorbance and $Li_2O_2$ concentrations.

Since the purity of commercial $Li_2O_2$ is only 95%, we synthesized $Li_2O_2$ powder with a purity over 99% for the calibration of UV-vis intensity with $Li_2O_2$ concentrations. The specific synthesis method is as follows:

10.45 g LiOH $H_2O$ was first added to 200 ml methanol with vigorous stirring for 24 hours. Then 42.5 g $H_2O_2$ (30% water solution) was injected to the solution. Stirring was continued for 1 hour to obtain a suspension, which was subsequently filtered. All the above operations were carried out in an Ar-filled glove box to exclude the effect of $CO_2$. Afterwards, the obtained sample was dried under vacuum at 110℃ for 24 hours to obtain $Li_2O_2$ powder.

A purity exceeding 99% was achieved by titration of the chemically synthesized $Li_2O_2$ powder with $KMnO_4$ solution. The principle of the titration is as follows:

$$Li_2O_2 + H_2SO_4 = Li_2SO_4 + H_2O_2$$

$$2KMnO_4 + 5H_2O_2 + 3H_2SO_4 = K_2SO_4 + 2MnSO_4 + 8H_2O + 5O_2$$

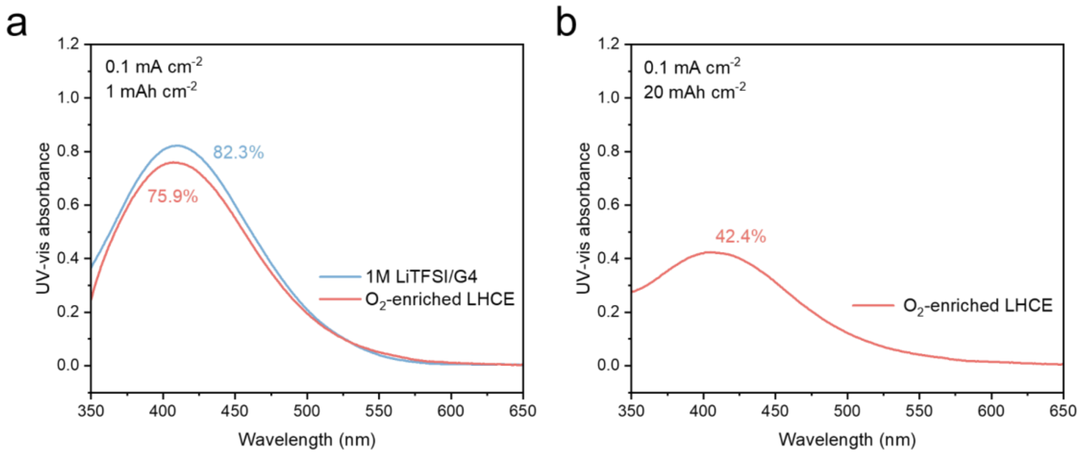


Figure S11. (a) The UV-vis spectra of the $TiOSO_4$ solutions with the cathode abstracted from LOBs with different electrolytes. The discharge capacity is 1 mAh $cm^{-2}$. (b) The UV-vis spectra of the $TiOSO_4$ solutions with the cathode abstracted from LOBs with the $O_2$-enriched LHCE. The discharge capacity is 20 mAh $cm^{-2}$.

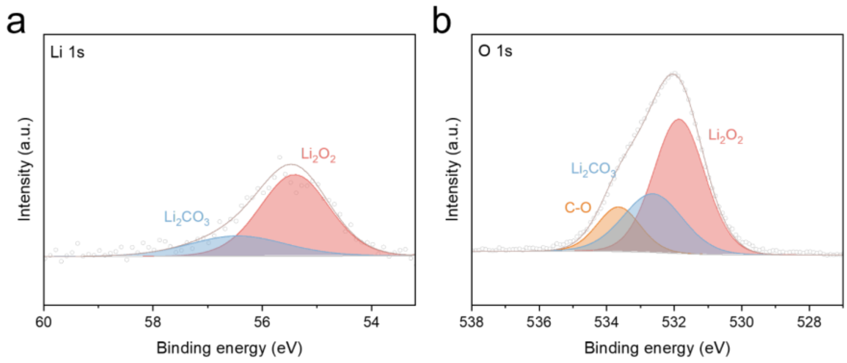


Figure S12. XPS spectra of the discharged cathodes with $O_2$-enriched LHCE: (a) Li 1s, (b) O 1s.

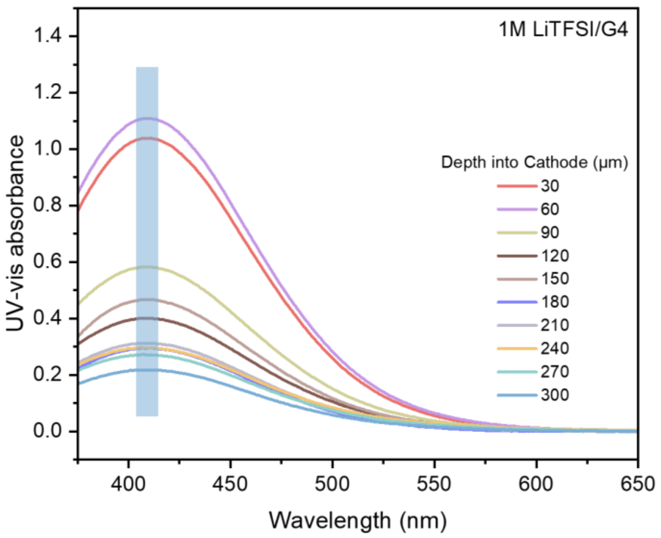


Figure S13. The UV-vis spectra of the $TiOSO_4$ solutions with the discharged cathode microtomed to 30 μm slices. The electrolyte is 1M LiTFSI/G4.

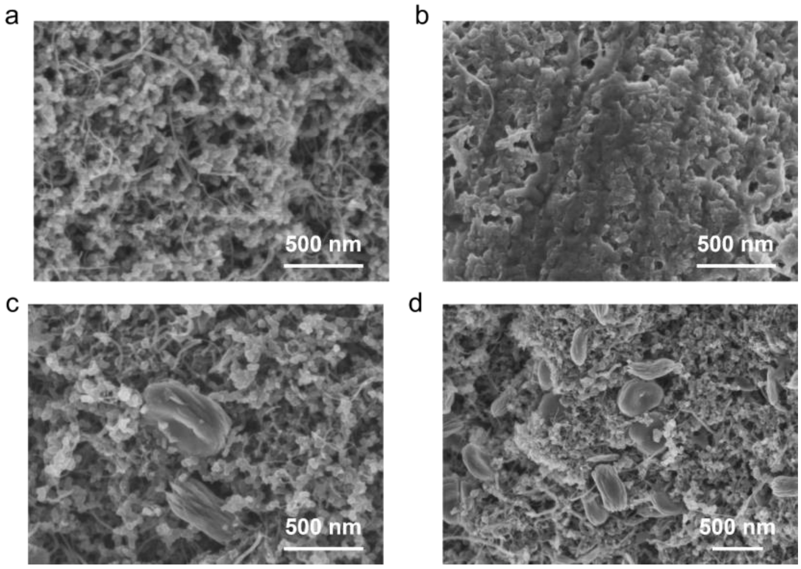


Figure S14. SEM images of the (a) pristine cathode, (b) the cathode/separator interface of cathodes discharged to a capacity of 10 mAh $cm^{-2}$ obtained from LOB with 1 M LiTFSI/G4, and (c)-(d) the cathode/separator interface of cathodes discharged to a capacity of 30 mAh $cm^{-2}$ obtained from LOB with the $O_2$-enriched LHCE.

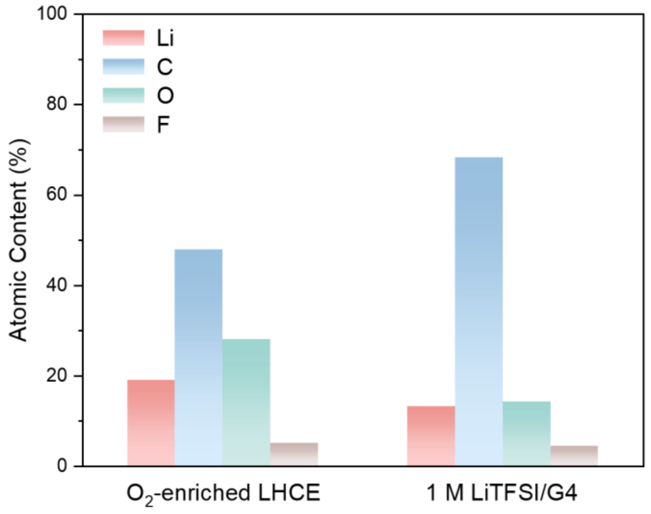


Figure S15. The element composition on the surface of the Li metal anode discharged with different electrolytes.

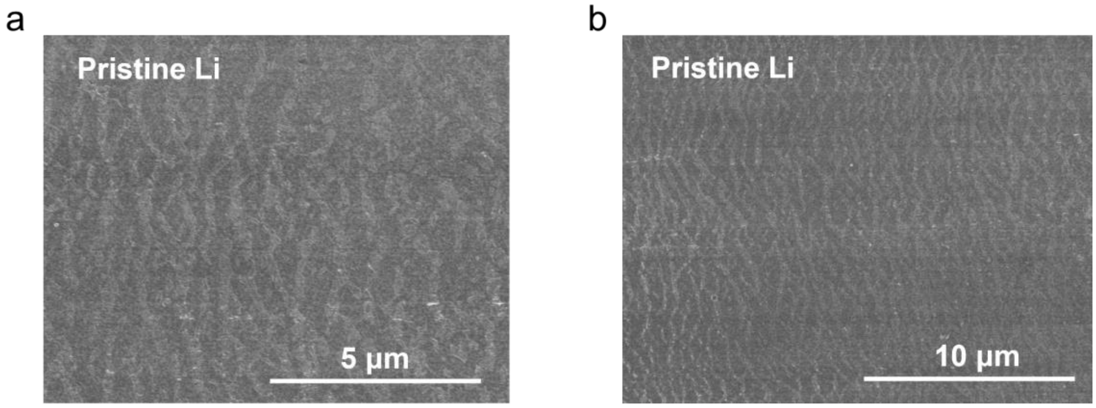


Figure S16. (a-b) SEM images of the pristine Li metal anode.

| Component | Size (cm) | Thicknesses (μm) | Areal Mass ($mg\ cm^{-2}$) |
|---|---|---|---|
| Carbon Paper | 6*8 | 65 | 1.15±0.01 |
| Cathode | 6*8 | 345±15 | 11.25±0.15 |
| Li Metal | 6*8 | 197.5±1.0 | 11.46±0.03 |
| Cu-PET Foil | 6*8 | 6 | 2.10±0.03 |
| Anode | 6*8 | 399±2.0 | 25.00±0.05 |

Figure S17. Parameters of the components in the Li-$O_2$ pouch cell.

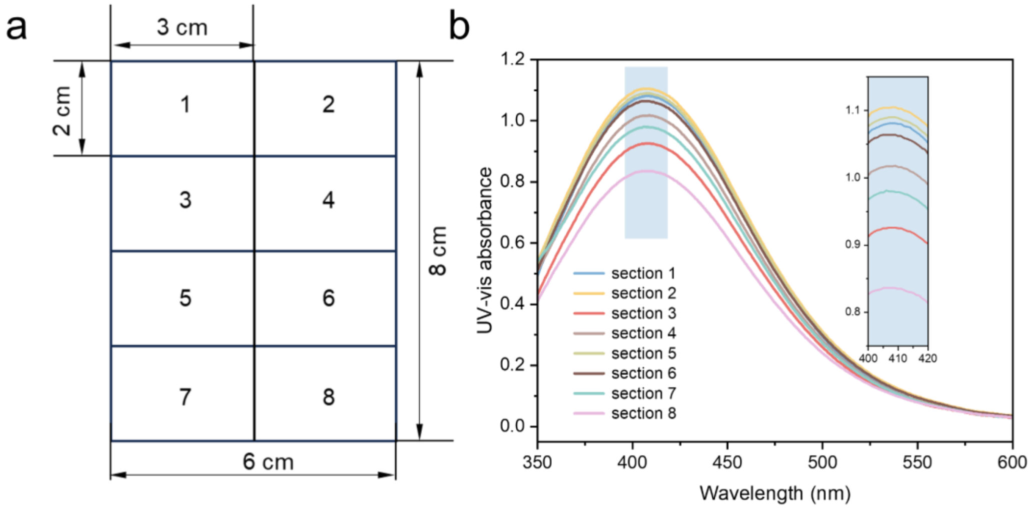


Figure S18. (a) Schematic diagram of a discharged Li-$O_2$ pouch cell cathode uniformly cut into 8 sections. (b) The UV-vis spectra of the $TiOSO_4$ solutions with the discharged Li-$O_2$ pouch cell cathode cut into 8 sections.

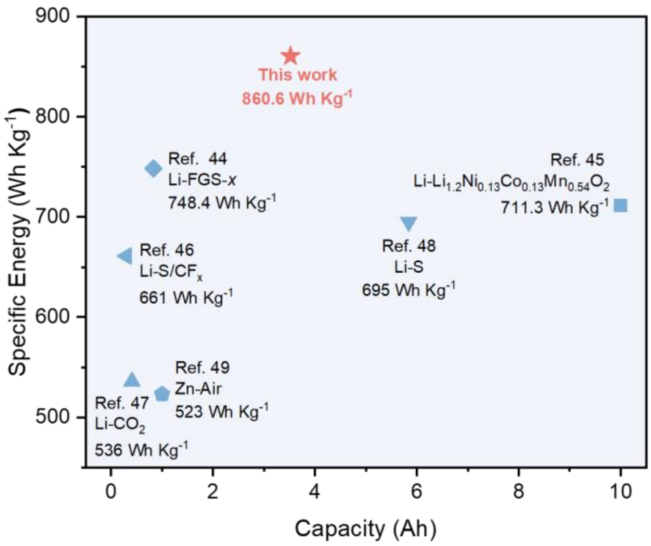


Figure S19. Electrochemical performance comparison of reported research on pouch cells of other advanced battery systems.

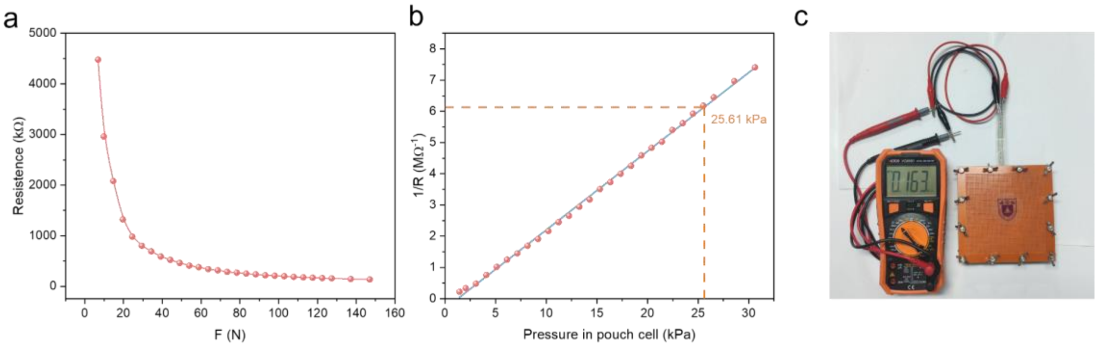


Figure S20. (a) The measured resistance to the applied force of the MFSR. (b) The fitted standard response curve of resistance to pressure of the MFSR. (c) Digital image of the pressure measuring of the Li-$O_2$ pouch cell using the MFSR.

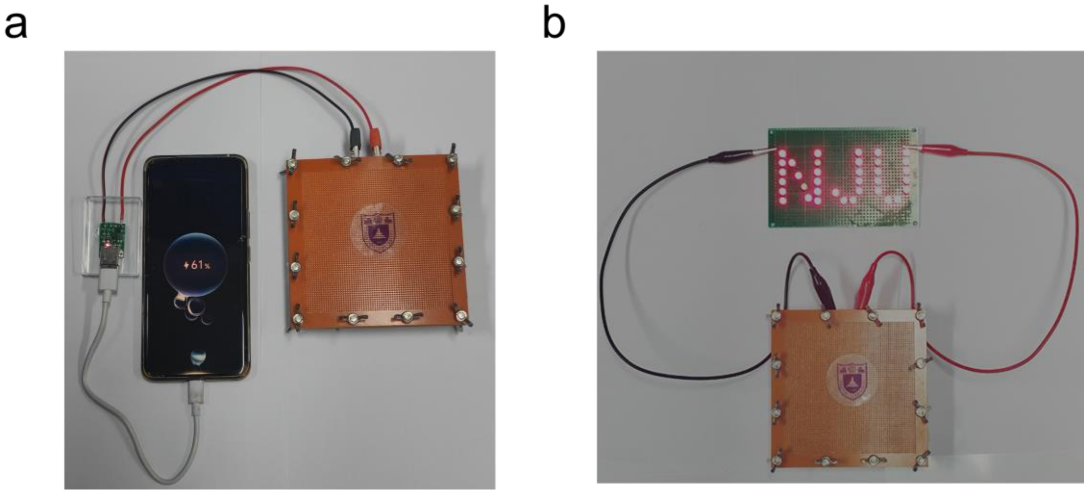


Figure S21. Photograph of the Li-$O_2$ pouch cell (a) charging a smartphone, (b) powering a LED light board.

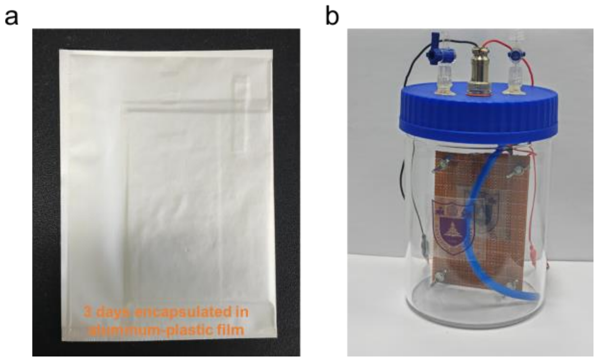


Figure S22. (a) Digital photograph of the Li-$O_2$ pouch cell vacuum encapsulated in an aluminum-plastic film. (b) Digital photograph of electrochemical performance test device for Li-$O_2$ pouch cells.

Table S1 Properties of various fluorinated ether diluents

| | Boiling point (℃) | Density (g ml$^{-1}$) | Flash point (℃) | Cost* |
|---|---|---|---|---|
| tris(2,2,2-trifluoroethyl)orthoformate (TTFEO) | 144 | 1.46 | 59 | high |
| 1,1,2,2-Tetrafluoroethyl-2,2,3,3-tetrafluoropropylether (TTE) | 92 | 1.53 | 27 | low |
| 1H,1H,5H-octafluoropentyl-1,1,2,2-tetrafluoroethyl ether (OTE) | 133 | 1.53 | 45 | low |
| bis(2,2,2-trifluoroethyl) ether (BTFE) | 63 | 1.41 | 1.7 | high |
| ethylene glycol bis(1,1,2,2-tetrafluoroethyl) ether (EGBTFE) | 141 | 1.41 | 45 | low |

*The cost of each diluent was estimated by a comprehensive comparison of reagent prices on the official websites of Sigma-Aldrich, Alfa Aesar, and Apollo.

Table S2 The $O_2$ solubility of electrolytes reported in the published literature.

| Electrolyte | $O_2$ solubility |
|---|---|
| TFEO-LHCE [1] | 0.24 cc $O_2$/cc (10.7 mM) |
| 1 M LiTFSI/PC-EC (1:1 wt) [2] | 0.18 mM |
| PC-EC (1:1 wt) [3] | 0.18 mM |
| DMSO [3] | 0.19 mM |
| DMA/M3 [4] | 0.13 mM |
| 0.5 M EMITFSI/DME [5] | 1.9 mM |
| 1 M LiTFSI/G4 with 2% TCCF [6] | 0.25 mM |
| **This work** | **10.68 mM** |

Table S3 Comparison of the electrochemical performance of published Li-$O_2$ pouch cells.

| | Specific energy (Wh $kg^{-1}$) | Capacity (Ah) | Current density (mA $cm^{-2}$) | Weight based |
|---|---|---|---|---|
| Ref. 33 | 757.5 | 0.82 | 0.05 | anode, cathode, separator, and electrolyte |
| Ref. 34 | 768.5 | 8.7 | 0.05 | total pouch cell |
| Ref. 35 | 362 | 1.19 | 0.05 | total pouch cell |
| Ref. 36 | 680 | 1.07 | 0.09 | anode, cathode, separator, and electrolyte |
| Ref. 37 | 536 | 0.51 | 0.28 | total pouch cell |
| Ref. 38 | 302.5 | 4.37 | 8.8 | total pouch cell |
| Ref. 39 | 408.5 | 0.077 | 0.26 | total pouch cell |
| Ref. 40 | 513.5 | 5.5 | | total pouch cell |
| Ref. 41 | 523.1 | Approx. 0.61 | | total pouch cell |
| Ref. 42 | 441.6 | Approx. 0.86 | | total pouch cell |
| Ref. 43 | 1214 | | | anode, cathode, and electrolyte |
| This work | 860.6 | 3.52 | 0.1 | total pouch cell |

Table S4 Comparison of the electrochemical performance of published pouch cells of other advanced battery systems.

| | Specific energy ($Wh\ kg^{-1}$) | Capacity (Ah) | Weight based |
|---|---|---|---|
| Ref. 44 | 748.4 | 0.83 | total pouch cell |
| Ref. 45 | 711.3 | 10 | total pouch cell |
| Ref. 46 | 661 | 0.3 | total pouch cell |
| Ref. 47 | 536 | 0.411 | total pouch cell |
| Ref. 48 | 536 | 0.411 | total pouch cell |
| Ref. 49 | 695 | 5.84 | total pouch cell |
| This work | 860.6 | 3.52 | total pouch cell |

## REFERENCES


1. Kwak W-J, Lim H-S, Gao P *et al.* Effects of fluorinated diluents in localized high-concentration electrolytes for lithium–oxygen batteries. *Adv Funct Mater* 2021; **31**: 2002927.

2. Xu W, Xiao J, Zhang J *et al.* Optimization of nonaqueous electrolytes for primary lithium/air batteries operated in ambient environment. *J Electrochem. Soc* 2009; **156**: A773.

3. Xu W, Hu J, Engelhard MH *et al.* The stability of organic solvents and carbon electrode in nonaqueous Li-$O_2$ batteries. *J Power Sources* 2012; **215**: 240-247.

4. Yang D-Y, Du J-Y, Yu Y *et al.* Stable lithium oxygen batteries enabled by solvent-diluent interaction in N,N-dimethylacetamide-based electrolytes. *Angew Chem Int Ed* 2024; **63**: e202403432.

5. Yuan R, Tan C, Zhang Z *et al.* Topological engineering electrodes with ultrafast oxygen transport for super‐power sodium‐oxygen batteries. *Adv Mater* 2024; **36**: 2311627.

6. Wang D, Zhang F, He P *et al.* A versatile halide ester enabling Li-anode stability and a high rate capability in lithium–oxygen batteries. *Angew Chem Int Ed* 2019; **58**: 2355-59.
